# Supplementary material for: Hidden patterns of codon usage bias across kingdoms
Source: J R Soc Interface. 2020 Feb 19;17(163):20190819. doi: 10.1098/rsif.2019.0819 (PMC7061699; doi:10.1098/rsif.2019.0819)
Supplement: Supporting Information [file rsif20190819supp1.pdf]

Hidden patterns of codon usage bias across kingdoms

Supporting Information

Deng Yun, Jeremie Kalfon, Dominique Chu, Tobias von der Haar

1 Theory

1.1 Beanbag model

Beanbag models assume that selection acts at the level of codons. Assigning codons to a particular site can be thought of as selecting beans from a bag. Each time a codon is picked, the choice between the codons / bags is made with a fixed probability that does not depend on the previous choices made. For each codon assignment there will be a probability p_i that the i-th codon is chosen among all possible $|C^{\mathcal{A}}|$ codons $C_1^{\mathcal{A}}, \dots, C_{|C^{\mathcal{A}}|}^{\mathcal{A}}$ for amino acid A. This type of selection procedure is generally known to lead to a multinomial distribution of codon subsequences. Specifically, the probability to observe a particular gene that has $L = L^{\mathcal{A},g}$ occurrences of a particular amino acid $\mathcal{A}$ with $m := |C^{\mathcal{A}}|$, and k_i occurrences of the i-th codon is given by

$$P = \frac{L!}{k_1! \cdots k_m!} q_1^{k_1} \cdots q_m^{k_m} \tag{1}$$

Here q_i is the global codon usage bias of the i-th codon. In the special case when there are only two codons in the amino acid, i.e. $m = 2$ then this probability reduces to the binomial distribution. We set $k := k_1$, $k_2 = L - k$, $p := q$ and $p_2 = 1 - q$.

$$P = \binom{L}{k} q^k (1-q)^{L-k} \tag{2}$$

1.1.1 Mixture models

A variant of the codon model is the multinomial mixture model, whereby selection still happens at the level of the individual subsequences, but the probability to select a codon depends on the particular subsequence. If we assume that there are M different groups of genes that each share the same selection pressure and the fraction of genes belonging to group i is F_i then we can calculate the expected frequencies of genes as follows (for the case of only 2 codons):

$$\begin{aligned} \mathcal{L}(k) &:= F_1 \binom{L}{k} \left(q_1^k (1-q_1)^{L-k}\right) + F_2 \binom{L}{k} \left(q_2^k (1-q_2)^{L-k}\right) + \dots F_M \binom{L}{k} \left(q_M^k (1-q_M)^{L-k}\right) \\ &= \binom{L}{k} \langle q_i^k (1-q_i)^{L-k} \rangle_F. \end{aligned}$$

Here the brackets indicate an ensemble average over the M classes. The empirical probability to observe a subsequence with k codons of type one is then given by

$$P_k = \frac{\mathcal{L}(k)}{\sum_{i=1}^{L} \mathcal{L}(i)}. \tag{3}$$

As the number of classes M grows, the probability P_k approaches the uniform probability distribution, since

$$\int_0^1 q_1^k (1-q_1)^{L-k} dq = \frac{1}{N+1} \binom{N}{k}.$$

1.2 Codon usage bias as a random walk

An alternative to the beanbag model is a model where not individual codons are selected, but specific subsequences. As an extreme example, consider the scenario where there is no underlying global codon usage bias at all and all codons occur equally often across the genome, but within a particular gene all codons for an amino acid $\mathcal{A}$ are strictly always the same. In this case the probability $p_i^{\mathcal{A}} = 1/|C^{\mathcal{A}}|$, but the probability to observe a particular subsequence would not follow the binomial distribution. In fact, there would be only $C^{\mathcal{A}}$ different subsequences of codons for each amino acid of a particular gene.

For the derivation of the full model, it is convenient to consider codon evolution as a random walk. We consider here a model whereby the position of the codons does not matter, and we only care about the number of codons in a particular subsequence. This analysis remains thus insensitive to correlations of codon usage within genes.

For simplicity, we focus here exclusively on amino acids with 2 codons, yet extensions to more codons are straightforward. In the case of 2 codons, codon evolution can be represented as a 1D random walk in discrete space and continuous time. The number of sites is $L^{\mathcal{A},g}$. Each site corresponds to a particular subsequence composition, i.e. a pair $(k_1^{\mathcal{A},g}, L^{\mathcal{A},g} - k_2^{\mathcal{A},g})$, which defines a state. In the case of 2 codons the state is entirely characterised by k_1. States are connected (i.e. accessible to the random walker) when a single synonymous codon change is sufficient to get from one state to the other. In the case of 2 codons this means that there are two states with one connected state $(0, L^{\mathcal{A},g})$ and $(L^{\mathcal{A},g}, 0)$). All other states are connected to two other states states, such that k_1 is connected to $(k_1 - 1, L^{\mathcal{A},g} - k_1 + 1)$ and $(k_1 + 1, , L^{\mathcal{A},g} - k_1 + 1)$. When there are more than 2 codons, then each state is connected to more than two states.

A transition from one state to another always involves that a codon of a particular type is changed to a codon of a different type. The rate of such an event is proportional to the number of codons of the type that is lost. For example, the rate of transitions where codon 2 is converted to a codon of type 1 is proportional to k_2.

$$r(k_1, k_2, \ldots \to k_1 + 1, k_2 - 1, \ldots) \sim k_2 \tag{4}$$

In order to derive a model, we now take a conceptual leap and (i) model each subsequence as a site of the random walk that has a given energy E_i, where the index i counts the numbers of codons of type 1 (which we choose arbitrarily). This energy is, at least for the time being, an entropic energy in that it is the (conceptual) consequence of the number of subsequence configurations that have k_1 codons of type 1. This number is lowest when all codons are of the same type and highest when all codons are used exactly equally often. (ii) We posit that the random walker that moves between the sites is in contact with a large heat-bath that remains at a fixed temperature T. This temperature bath exchanges energy with the walker, thus enables it to transition to sites with higher energy, as well as lower energy while extracting/adding energy to the heat bath. We stress here that this idea of energy and temperature are merely conceptual devices and should not be confused with the actual physical temperature that is experience by organisms.

Having made this conceptualisation, we can now apply well established concepts from stochastic thermodynamics [1] to this problem in order calculate the long-term equilibrium probabilities $\pi(k_1, k_2, \ldots)$ for various configurations. To do this we impose that the system obeys the detailed balance condition, which means that in equilibrium there are no net-flows of probability between any two states that are connected.

$$\pi(k_1, k_2, \ldots) r(k_1, k_2, \ldots \to k_1 + 1, k_2 - 1, \ldots) = \pi(k_1 + 1, k_2 - 1, \ldots) r(k_1 + 1, k_2 - 1, \ldots \to k_1, k_2, \ldots) \tag{5}$$

Here $r(k_1, k_2, \ldots \to k_1 + 1, k_2 - 1, \ldots)$ is the rate of a mutation from any of the k_1 codons of type 1 to codon 2. This implies that

$$\frac{\pi(k_1, k_2, \ldots)}{\pi(k_1 + 1, k_2 - 1, \ldots)} = \frac{k_1 + 1}{k_2} \tag{6}$$

We also know that the occupation probabilities of a random walker of the type described here obeys the Boltzmann distribution in equilibrium. This means that the probability to find the walker in a given configuration depends on the energy of the configuration $E(k_1, k_2, \ldots)$ in the following way:

$$\pi(k_1, k_2, \ldots) = \frac{1}{Z} \exp\left(-\frac{E(k_1, k_2, \ldots)}{k_B T}\right). \tag{7}$$

Since we are not interested here in specific units, we will henceforth set the Boltzmann constant $k_B = 1$.

The energy is *a priori* unknown, but we postulate that the local detail balance condition is fulfilled [2]:

$$T \ln\left(\frac{r_+}{r_-}\right) = \Delta E, \tag{8}$$

where r_+ and r_- are the forwards and backwards transition rates respectively. This relationship then implies a dependence between the two rates, namely:

$$r_+ = r_- \exp\left(\frac{\Delta E}{T}\right) \tag{9}$$

1.2.1 Calculation of the energy of a specific codon in the completely unbiased beanbag model.

We first calculate the energies associated with a model where there is no selection pressure at all and codons perform a random walk only. This is the beanbag model where all codons have the same probability of being chosen. To calculate the energies of each subsequence, we start from a subsequence where all L codons are of type 1. We define this state as having energy $E_0 = 0$. A mutation can reach the next state (1) by changing one of the L codons of type 1 to a codon of type 2. When there are no selection forces then this happens with a rate proportional to L. From this state (1), the system can then move further to state (2). This happens now with a rate proportional to $L-1$. Alternatively, with a rate proportional to 1 it can move back to state (0). Altogether, the following transitions are thus possible:

$$(L,0) \underset{1}{\overset{L}{\rightleftharpoons}} (L-1,1) \underset{2}{\overset{L-1}{\rightleftharpoons}} \cdots \underset{k}{\overset{L-k+1}{\rightleftharpoons}} (L-k,k) \underset{k+1}{\overset{L-k}{\rightleftharpoons}} (L-k-2,k+2) \cdots \underset{L}{\overset{1}{\rightleftharpoons}} (0,L) \tag{10}$$

Using the local detailed balance condition (eq. 8) and because the energy associated with state (0) is by construction $E_0 := 0$, the energy difference between state (0) and state (1) is $\Delta E_1 = \ln(L/1)$. Generally, the energy difference ΔE_i between state (i) and state $(i-1)$ is given by $\Delta E_i = \ln\left((L-i+1)/i\right)$. Consequently, the energy of state (k) is then:

$$\begin{aligned} E_k &= \sum_{i=1}^{k} \Delta E_i = -T \ln\left(\prod_{i=1}^{k} \frac{L-i+1}{i}\right) = \\ &= -T \ln\left(\frac{L!}{(L-k)!k!}\right) = -T \ln\binom{L}{k}. \end{aligned} \tag{11}$$

This entails that the (Boltzmann-)probability

$$P(E_k) = \frac{\exp\left(-\frac{E_k}{T}\right)}{\sum_i \exp\left(-\frac{E_i}{T}\right)}$$

to observe a specific configuration k is proportional to the binomial coefficient, which indicates that this system is distributed according to the binomial distribution eq. 2 with $p = 1/2$. As such it represents a model with no selection pressure at the level of sequences.

1.2.2 The binomial distribution with $q \neq 1/2$.

We now establish an energy model of the binomial distribution when there is an underlying bias to the mutations. In this case then the model is (c.f. eq. 10):

$$(L,0) \underset{1\cdot(1-q)}{\overset{Lq}{\rightleftharpoons}} (L-1,1) \underset{2(1-q)}{\overset{(L-1)q}{\rightleftharpoons}} \cdots \underset{k(1-q)}{\overset{(L-k+1)q}{\rightleftharpoons}} (L-k,k) \underset{(k+1)(1-q)}{\overset{(L-k)q}{\rightleftharpoons}} (L-k-2,k+2) \cdots \underset{L(1-q)}{\overset{1\cdot q}{\rightleftharpoons}} (0,L) \tag{12}$$

Following the same reasoning as above, we can establish the energy differences:

$$
\begin{aligned}
\hat{E}_0 &= 0 \\
\Delta\hat{E}_1 &= -T\ln\left(\frac{Lq}{1\cdot(1-q)}\right) \\
\Delta\hat{E}_2 &= -T\ln\left(\frac{(L-1)q}{2(1-q)}\right) \\
\Delta\hat{E}_k &= -T\ln\left(\frac{(L-k+1)q}{k(1-q)}\right) \qquad (13)
\end{aligned}
$$

Hence, it follows that the energy $\hat{E}_k$ is given by:

$$
\begin{aligned}
\hat{E}_k &= -\ln\left(\frac{(L-k+1)!}{(L-k)!k!}\cdot\frac{q^k}{(1-q)^k}\right) \\
&= -\ln\binom{L}{k} + \ln\left(\frac{(1-q)^k}{q^k}\right) \\
&= E_k + \ln\left(\frac{(1-q)^k}{q^k}\right) \qquad (14)
\end{aligned}
$$

The corresponding Boltzmann distribution can be shown to be the binomial distribution eq. 2 with $p = q$.

1.2.3 Asymmetric bias

For completeness we also consider the case where the transition rates are biased in one direction only. To this end we modify the rates such that there is a constant bias C into one direction. The random walk is then modified as follows:

$$
(L,0) \underset{C}{\overset{L}{\rightleftharpoons}} (L-1,1) \underset{2C}{\overset{(L-1)}{\rightleftharpoons}} \cdots \underset{kC}{\overset{(L-k+1)}{\rightleftharpoons}} (L-k,k) \underset{(k+1)C}{\overset{(L-k)}{\rightleftharpoons}} (L-k-2,k+2) \cdots \underset{LC}{\overset{1}{\rightleftharpoons}} (0,L) \qquad (15)
$$

Following the same steps as above, we can calculate energies for this model.

$$
\begin{aligned}
E'_k &= \sum_{i=1}^{k}\Delta E'_i = -T\ln\left(\prod_{i=1}^{k}\frac{(L-i+1)^\gamma}{i^\gamma C}\right) = \\
&= -T\ln\left(\frac{L!}{(L-k)!k!}\right) + k\ln(C) = E_k + ln(C^k) \qquad (16)
\end{aligned}
$$

The average codon usage ϵ is then given by

$$
q = \frac{A}{LZ} = \frac{1}{C+1} \qquad (17)
$$

1.3 Sequence selection models

We now further modify the rates of the model and assume that there is a selection pressure on the genome such that the rate with which codon 2 mutates to codon 1 and *vice versa* is now no longer proportional to the number $N-k+1$ and k of codon 2, but proportional to a power of this number. This then changes the above model, as follows:

$$
(L,0) \underset{1^\gamma}{\overset{L^\gamma}{\rightleftharpoons}} (L-1,1) \underset{2^\gamma}{\overset{(L-1)^\gamma}{\rightleftharpoons}} \cdots \underset{k^\gamma}{\overset{(L-k+1)^\gamma}{\rightleftharpoons}} (L-k,k) \underset{(k+1)^\gamma}{\overset{(L-k)^\gamma}{\rightleftharpoons}} (L-k-2,k+2) \cdots \underset{L^\gamma}{\overset{1^\gamma}{\rightleftharpoons}} (0,L) \qquad (18)
$$

The energies then become:

$$
\begin{aligned}
\tilde{E}_k &= \sum_{i=1}^{k}\Delta\tilde{E}_i = -T\ln\left(\prod_{i=1}^{k}\frac{(L-i+1)^\gamma}{i^\gamma}\right) = \\
&= -T\gamma\ln\left(\frac{L!}{(L-k)!k!}\right) = \gamma E_k \qquad (19)
\end{aligned}
$$

We conclude that, if the transition rates are as in the unbiased case, but modified by the exponent, then the energies of the model remain unaffected, but the temperature of the system changes according to γ. Note that in this model, on average, the number of codons of type 1 will always be the same as the number of codons of type 2. Thus no global codon usage bias would occur. Selection manifests itself in the way that codons are distributed across subsequences, which is quantified by the inverse temperature γ.

Finally, we now consider the most general model with different exponents to the left and to the right to arrive at the full model.

$$(L,0) \underset{1^\gamma}{\overset{L^\xi}{\rightleftharpoons}} (L-1,1) \underset{2^\gamma}{\overset{(L-1)^\xi}{\rightleftharpoons}} \cdots \underset{k^\gamma}{\overset{(L-k+1)^\xi}{\rightleftharpoons}} (L-k,k) \underset{(k+1)^\gamma}{\overset{(L-k)^\xi}{\rightleftharpoons}} (L-k-2,k+2) \cdots \underset{L^\gamma}{\overset{1^\xi}{\rightleftharpoons}} (0,L) \tag{20}$$

This changes the energies of the model in the following way:

$$\begin{aligned} \bar{E}_k &= \sum_{i=1}^{k} \Delta \bar{E}_i = -T \ln \left(\prod_{i=1}^{k} \frac{(L-i+1)^\zeta}{i^\gamma} \right) \\ &= -T\xi \ln \binom{L}{k} + T(\gamma - \xi) \ln(k!) \\ &= \xi E_k + T(\gamma - \xi) \ln(k!) \end{aligned} \tag{21}$$

It is apparent that this case changes both the inverse temperature of the system and the expected number of codons of type 1 relative to the case of no selection. The corresponding Boltzmann distribution,

$$P(\bar{E}_k) = \frac{\xi E_k + T(\gamma - \xi) \ln(k!)}{\sum_i \left(\xi E_i + T(\gamma - \xi) \ln(i!)\right)} \tag{22}$$

is a mono-modal distribution. The mean of the distribution depends on the parameters ξ and γ. For certain choices of these parameters, $P(\bar{E}_k)$ can approximate a binomial distribution with some bias $p \neq 1/2$.

2 Further details on methods used

Fitting the data

Each gene in the clean sequence files was then split into (up to) 18 OCCs as follows: For each gene g and amino acid $\mathcal{A}$ in the dataset we found all codons that code for $\mathcal{A}$ and discarded all others. Thus, we reduced the gene g to an OCC of codons of length $L^{\mathcal{A},g}$.

For each OCC thus produced, a control coding OCC was generated by replacing each codon with a random synonymous codon (which could be the same as the one in the original OCC). The probability of choosing a random synonymous codon was biased according to the observed global codon frequency bias of the respective species and amino acid, such that in the control data the codons were distributed according to the multinomial distribution by construction.

For the results presented in the main text we limited the analysis to the 9 amino acids with only 2 codons because the calculation of $\mathcal{D}$ becomes statistically unreliable when OCC sets are too small, and this can generate problem in the analysis of amino acids encoded by more than two codons. The number of *possible* OCCs grows quickly with the number of codon possibilities, whereas the *actual* number of OCCs represented in a genome does not. As a consequence, there are fewer examples per configuration which increases the statistical error.

The empirical estimates for the parameters of the full model were obtained by fitting genomic data to eq. 6 as follows:

1. For each species we split up each gene into 18 OCCs (or 9 respectively when we focussed on the 2 codon amino acids).

2. We then discarded all OCCs with more than 15 or fewer than 5 codons.

3. From the remaining data, we calculated the normalised frequencies of each codon as the number of occurrences of each codon divided by the total number of codons in the subset. This provided an empirical estimate of the energy E_i.

4. Next, we fitted these empirical E_i values to the full model eq. 6, thus obtaining estimates for γ and ξ for each length, i.e. 11 different fits per amino acid.

5. Species-wide distances $\mathcal{D}$ were calculated by taking occurrence weighted averages over the individual fits of the species.

Fitting was done using the Maple 2018 "NonlinearFit" function. The initial estimates for γ and ξ were set to 1. If an initial fit resulted in a mean-residual > 0.0009999 then the fit was repeated with randomly chosen initial estimates. This was repeated up to 1000 times until a fit was found with a mean-residual < 0.00099999.

Definition of ambiguous region

Fig. 2c shows the residuals obtained from the full model versus the residuals obtained from the binomial model. If the former is low and the latter is high then the corresponding OCC is better explained by SLS than the beanbag model. However, there is an ambiguous region where the beanbag model fits equally well as the full model (or better). There are many ways to define such a region, and we chose the following conservative approach: We first fitted a straight line to the corresponding plot of the control data in fig. 2b. Then we decided that all points in the plot for the full model in fig. 2c that lie below this line are ambiguous.

3 Global temperatures

For each set of subsequences corresponding to a particular length and amino acid, we next define an empirical energy $\mathcal{E}$ as

$$\mathcal{E}_{k_1,\ldots,k_{|C^{\mathcal{A}}|}} := -\ln\left(\frac{n_{k_1,\ldots,k_{|C^{\mathcal{A}}|}}}{N}\right), \tag{23}$$

where $n_{k_1,\ldots,k_{|C^{\mathcal{A}}|}}$ is the number of occurrences of subsequences with configuration $k_1,\ldots,k_{|C^{\mathcal{A}}|}$ in the species and N is the number of subsequences of amino acid $\mathcal{A}$ with length $L = \sum_{i=1}^{|C^{\mathcal{A}}|} k_i$ in the species. In order to assign a global temperature to a species as a whole we plot the empirical energy $\mathcal{E}_{k_1,\ldots,k_{|C^{\mathcal{A}}|}}$ against the energy $\hat{E}_{k_1,\ldots,k_{|C^{\mathcal{A}}|}}$.

If the codons were distributed according to the multinomial distribution, then up to statistical errors

$$\mathcal{E}^L_{k_1,\ldots,k_{k_1,\ldots,k_{|C^{\mathcal{A}}|}}} = \hat{E}^L_{k_1,\ldots,k_{k_1,\ldots,k_{|C^{\mathcal{A}}|}}}$$

, i.e. plotting the empirical energies against $\hat{E}$ would result in a straight line with slope 1. In contrast, if the distribution of subsequences was distributed by a multinomial distribution with a higher temperature, then this plot would still result in a straight line, but with a slope corresponding to $1/T$.

We plotted the empirical energies $\mathcal{E}$ against $\hat{E}$ and found that the two energies are in good approximation, related via a straight line. Fig. 1a shows 4 examples of fungal species for the 9 amino acid with exactly 2 codons. In order to check the temperature of a species, we fitted $\mathcal{E}$ versus $\hat{E}$ to a straight line. The slope of this straight line can then be taken as an estimate for the inverse temperature for each of the 462 fungal species in our dataset, see fig. 1a. % par We found that the slopes are significantly and systematically different from 1 and significantly and systematically higher than slopes obtained from random controls; see fig. 1. Seen globally, the model is thus consistent with the full model and thus consistent with the hypothesis of sequence-level selection. At the same time, the significantly altered slopes are not consistent with the assumption of a purely codon-based selection, which would lead to a binomial distribution of data, and hence slopes close to 1.

While this global data on the slope can be used qualitatively to demonstrate the signature of sequence-level selection acting on codon usage, it cannot be reliably used to quantify this selection pressure. While the result that slopes systematically deviate from the random model is robust with respect to changes of the fitting protocol, the precise numerical values are not robust to such changes. In fig. 1b we reported slopes obtained by fitting the lines to the entire dataset. If instead fits are restricted to energies $\hat{E} < 5$, which would exclude the low probability points, then somewhat different numerical results would appear. Moreover, the

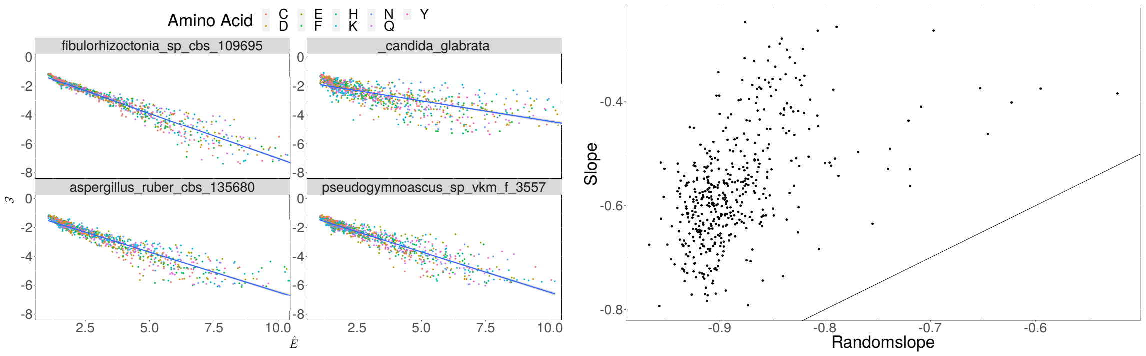

(a) (b)

Fig. 1: (a) Empirical energies $\mathcal{E}$ as a function of the energies $\hat{E}$ derived from the multinomial model assuming the global codon usage bias for each amino acid. Each plot combines data for 9 amino acids and 10 different lengths. Each point shows the theoretical $\hat{E}_k$ along the horizontal axis, denoting the logarithm of the binomial probability to observe a exactly k codons of type one in a subsequence of length $L^{\mathcal{A}}$, given the global codon usage bias for amino acid $\mathcal{A}$ in this species. The corresponding $\mathcal{E}$ is the logarithm of the observed fraction of occurrences of subsequences with k codons of type 1 amongst all subsequences of length $L^{\mathcal{A}}$. (b) The slopes obtained for all 462 fungal species plotted against slopes obtained from randomised genes where all codons were replaced by a random synonymous codons draw according to the global codon usage bias. If codons were distributed randomly across genes, then the points would cluster along the diagonal.

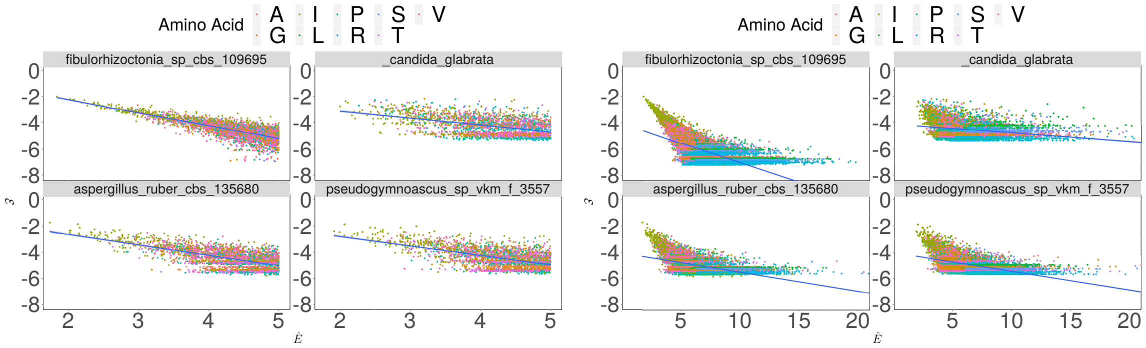

(a) (b)

Fig. 2: (a) Empirical energies $\mathcal{E}$ as a function of the energies $\hat{E}$ derived from the multinomial model assuming the global codon usage bias for each amino acid. Each plot combines data for the amino acids with more than 2 codons for length 5-15. Each point shows the theoretical $\hat{E}_k$ along the horizontal axis, denoting the logarithm of the multinomial probability to observe a exactly k_1 codons of type 1, k_2 codons of type 2, etc.., in a subsequence of length $L^{\mathcal{A}}$, given the global codon usage bias for amino acid $\mathcal{A}$ in this species. The corresponding $\mathcal{E}$ is the logarithm of the observed fraction of occurrences of subsequences with k codons of type 1 amongst all subsequences of length $L^{\mathcal{A}}$. The data (and the fit) is restricted to the subsequences where the theoretical distribution is greater than exp(-5). The blue line represent a fit to the data. (b) Same as 2a but the probabilities the data is not restricted to the high probability subsequences. The plot is dominated by noise.

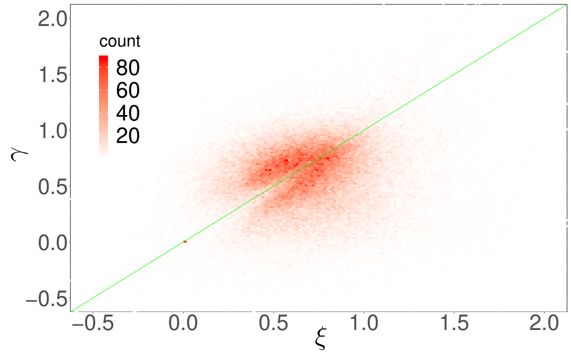

(a) Fungi

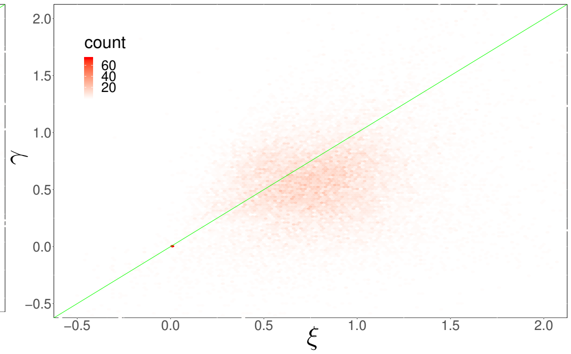

(b) Bacteria

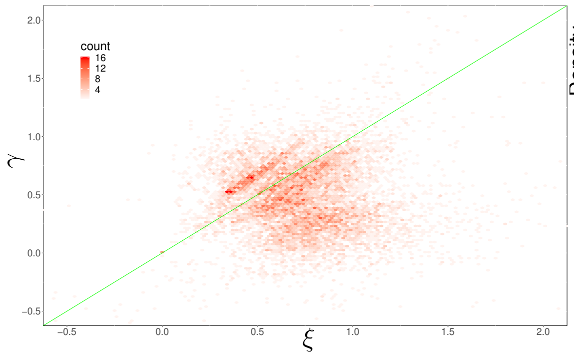

(c) Protists

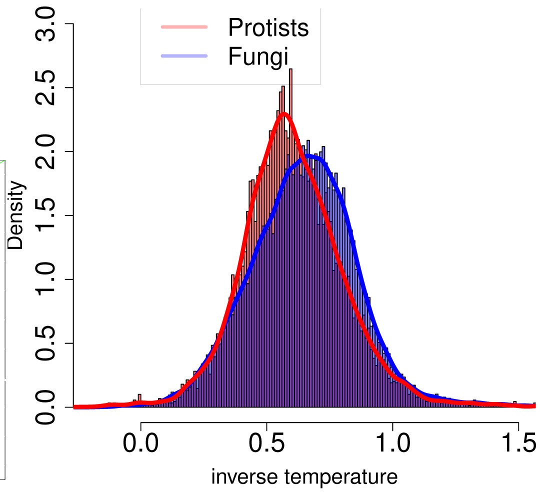

(d) Temperature: Protists and Fungi

Fig. 3: (a)-(c) The fitted values of parameters ξ and γ for each of the 2-codon amino acids for bacteria, fungi and protists. The graphs show heatplots that summarise the density of points in the area. Red indicates a high density of points. We are limiting ourselves to those amino acid subsequences that have a sub-length of 15. (d) The distribution of inverse temperatures of protists and fungi. There is considerable overlap between the two groups. Density estimates for each group are overlayed on the graph to aid the eye. It appears that fungi are somewhat cooler than protists. Bacteria would lie in-between protists and fungi, but are omitted to aid graph readability.

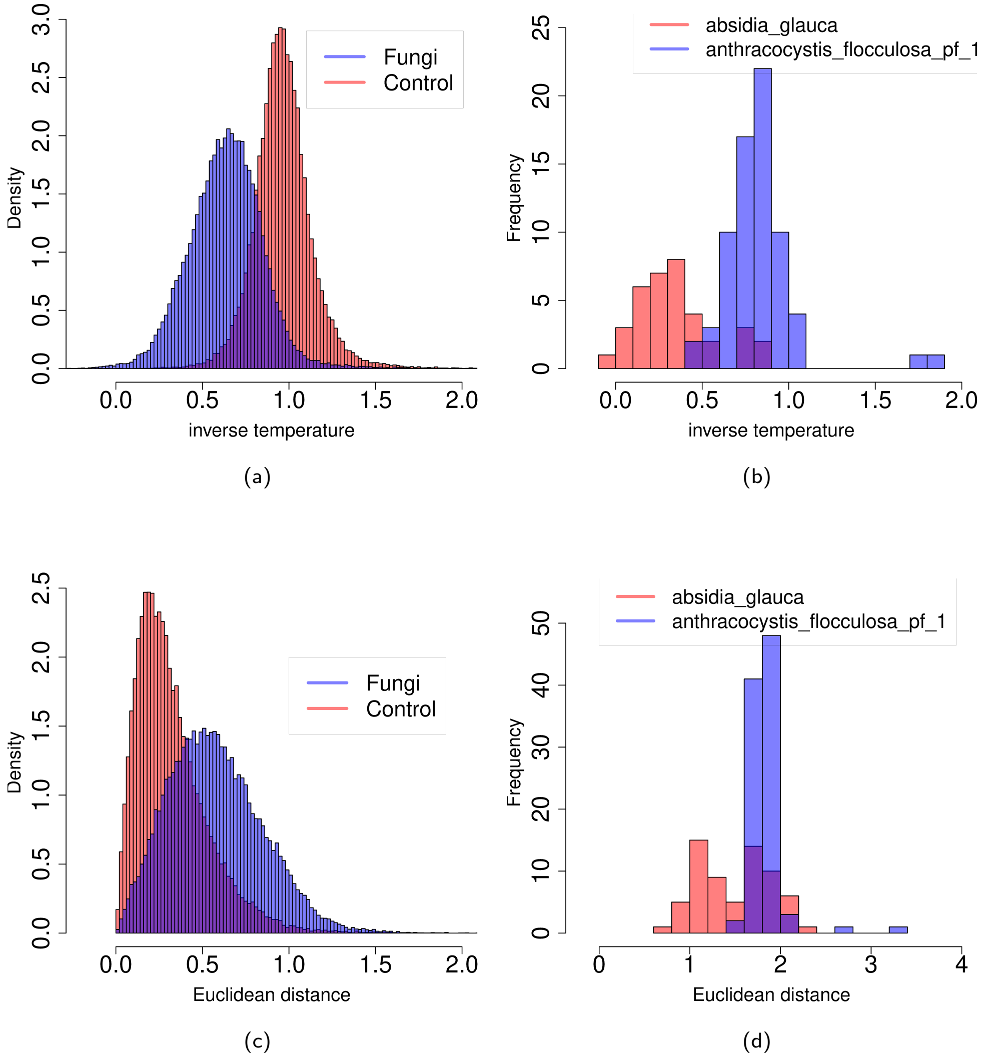

Fig. 4: (a) Distribution of inverse temperature in the fungal dataset showing all sub-length and all species. The control data peaks around an inverse temperature of 1, whereas the real data is distributed around a lower inverse temperature. (b) Distribution of inverse temperature for two different species. This shows the temperature for two species including all amino acids and is a subset of (a).4c The distribution of distances $\mathcal{D}$. The control data clearly has a smaller distance on the whole than the non-selection model, indicating that considering only the global codon usage bias underestimates the selection pressure. (d) Same data, but for two species only.

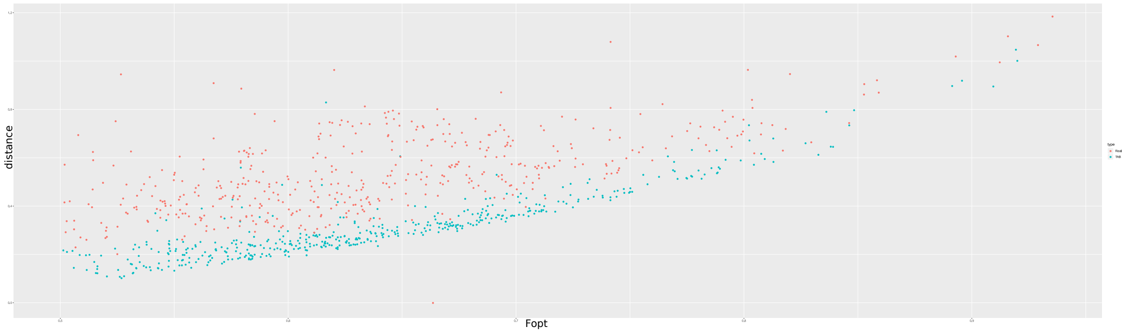

(a)

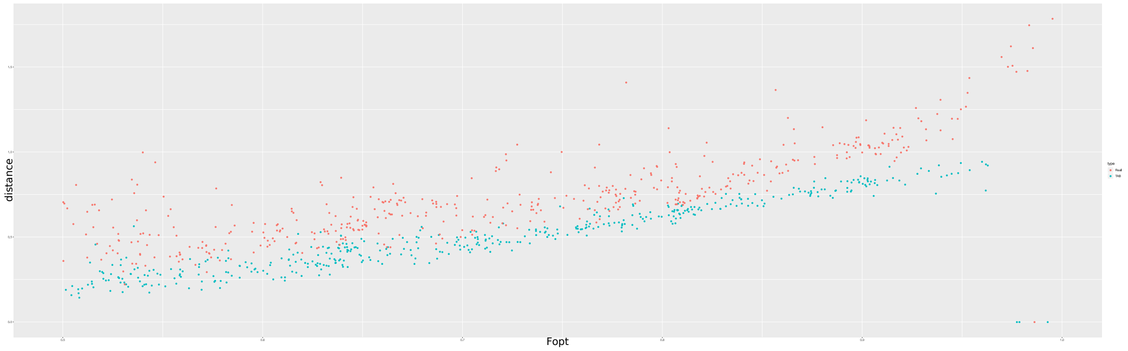

(b)

Fig. 5: (a) The distance $\mathcal{D}$ against Fopt for the actual genome (red) and a genome generated under the assumption of the beanbag model (blue). Each dot represents the distance for the amino acid F for a species in our fungal dataset. (b) Same, but for bacteria.

global slopes obtained thus are in no clear relationship with the summary statistics of the detailed fits of the full model to subsequences, even though they consist of exactly the same data.

We then repeated the same plot for the same species, but this time for the amino acids that have more than 2 codons. Fig. 2 demonstrates that these also lead to approximately straight lines, but only for the subset of high probability subsequences. For those we obtained slopes that were close to 1. When all subsequences are included then noise dominates and no meaningful fitting is possible; see fig. 2b.

4 Supplementary plots

Correlation for the parameter ξ for the fungal dataset. We include only those fits where the mean-residual was smaller than 0.0009999. The horizontal axis indicates the fitted value for the subsequence length of 15 and the vertical axis for the subsequences values $5 - 14$. The title indicates the Pearson correlation.

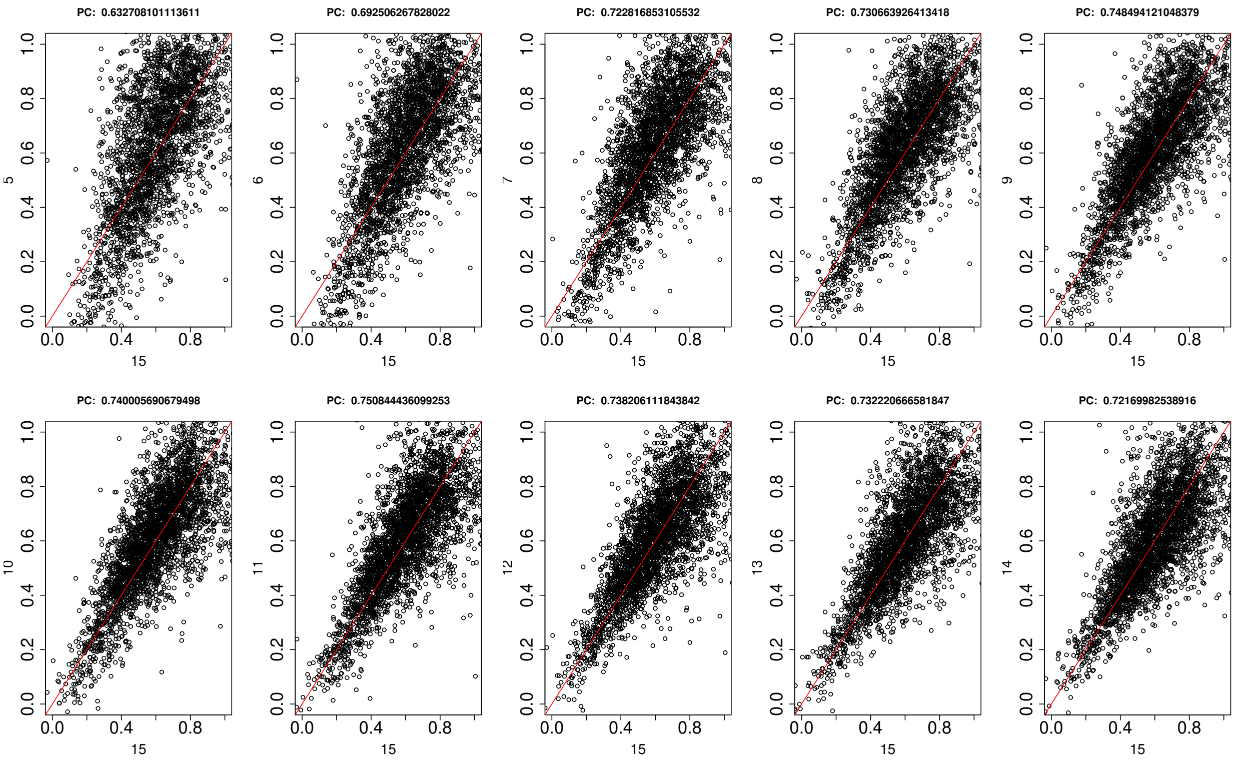

Same as above but for parameter γ.

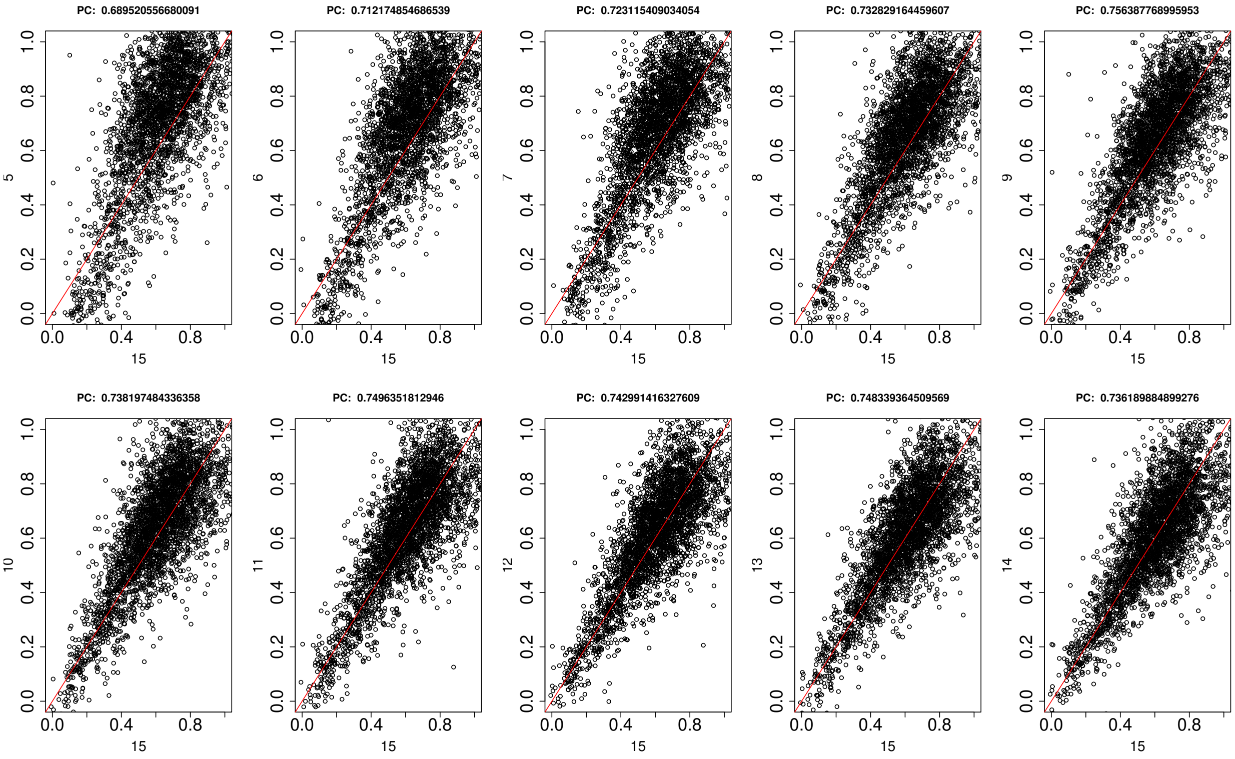
PC: 0.689520556680091
PC: 0.712174854686539
PC: 0.723115409034054
PC: 0.732829164459607
PC: 0.756387768995953
PC: 0.738197484336358
PC: 0.7496351812946
PC: 0.742991416327609
PC: 0.748339364509569
PC: 0.736189884899276
5
6
7
8
9
10
11
12
13
14
15

Same as above, but for the fits to the binomial distribution.

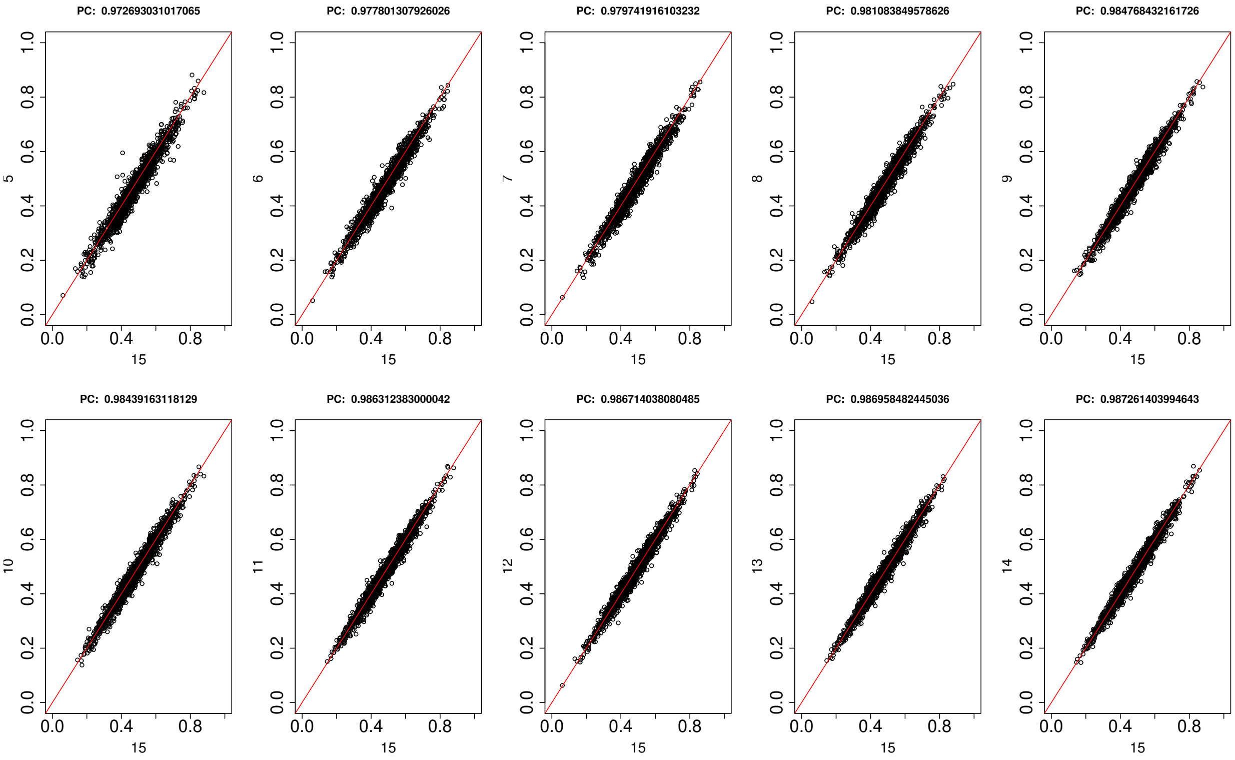

References

[1] U. Seifert. Stochastic thermodynamics, fluctuation theorems and molecular machines. *Reports on Progress in Physics*, 75(12):126001, 2012.

[2] G. Crooks. Nonequilibrium measurements of free energy differences for microscopically reversible markovian systems. *Journal of Statistical Physics*, 90(5/6):1481–1487, 1998.
